# Effect of Deposit Chemistry on the Stress Corrosion Cracking Susceptibility of CMSX-10 at 550°C and 700°C

**DOI:** 10.1007/s11085-025-10366-y

**Published:** 2025-12-20

**Authors:** F. Duarte Martinez, A. Syed, G. Gibson, J. Leggett, J. C. Mason-Flucke, J. R. Nicholls, S. Gray

**Affiliations:** 1 Department of Surface Engineering and Precision Institute, Cranfield University, Bedford, MK43 0AL UK; 2 Rolls-Royce Plc, PO Box 31, Derby, DE24 8BJ UK

**Keywords:** C-ring, Hot corrosion, Stress corrosion cracking, Superalloys

## Abstract

Turbine blades of aerogas turbines can be at risk of stress corrosion cracking (SCC) below 700 °C due to the effects of stress, sulphur-containing gases and deposits that are ingested into the turbine. Therefore, understanding the effect of different deposits on the SCC susceptibility of single-crystal nickel-based superalloys at different temperatures is crucial. This study investigated the effect of NaCl, sea salt and 80/20 mol% Na_2_SO_4_/K_2_SO_4_ on the SCC susceptibility of CMSX-10 at 550 °C and 700 °C. The results suggest that chlorine-containing salts play an important role in accelerating stress corrosion cracking at 550 °C, where the formation of HCl leads to the breaking down of the oxide and exposing the base alloy to a sulphidation environment. At 700 °C stress corrosion cracking is accelerated by the mix of sulphates that lead to reduced melting points, where the 80/20 mol% Na_2_SO_4_/K_2_SO_4_ has shown the highest susceptibility to SCC.

## Introduction

Aeroturbine blades are susceptible to stress corrosion cracking caused by their exposure to contaminants that enter the gas turbine, high-temperature sulphur-containing gases and the effect of stress. Some of the commonly observed deposits on turbine blades consist of a mix of NaCl, Na_2_SO_4_, CaSO_4_, K_2_SO_4_ and MgSO_4_, and their concentrations and ratios have a regional dependence and so does the SO_x_ concentration in the atmosphere [1, 2]. It is important to recognise that these different deposit compositions observed in engine applications can have different susceptibilities on the stress corrosion cracking behaviour of single-crystal nickel-based superalloys at different temperatures. Therefore, it is important to understand the effect of these individual deposits on accelerating corrosion attack and causing stress corrosion cracking in the 550–700 °C temperature range.

In previous work, NaCl, sea salt and the 80/20 mol% Na_2_SO_4_/K_2_SO_4_ salt mix have proved detrimental from a SCC point of view in nickel-based superalloys at temperatures of 550 °C [3–6] and at 700 °C [7]. However, the SO_x_ concentrations, magnitude of stresses and materials tested differ slightly in these studies, making the comparison between the salts challenging in terms of their susceptibility to SCC. In this work, a comparison between NaCl, sea salt and 80/20 mol % Na_2_SO_4_/K_2_SO_4_ is made in terms of the time it takes to initiate stress corrosion cracks and maximum crack depths observed on CMSX-10 after 100 h, 200 h and 500 h of exposure, at stresses of 450 MPa, at 550 °C and 700 °C and a 50 ppm SO_2_ – air environment (close to engine application [2]). An examination of the corrosion products and crack initiation mechanisms at 550 °C and 700 °C are discussed.

## Experimental Procedures

### Materials

**Fig. 1:**
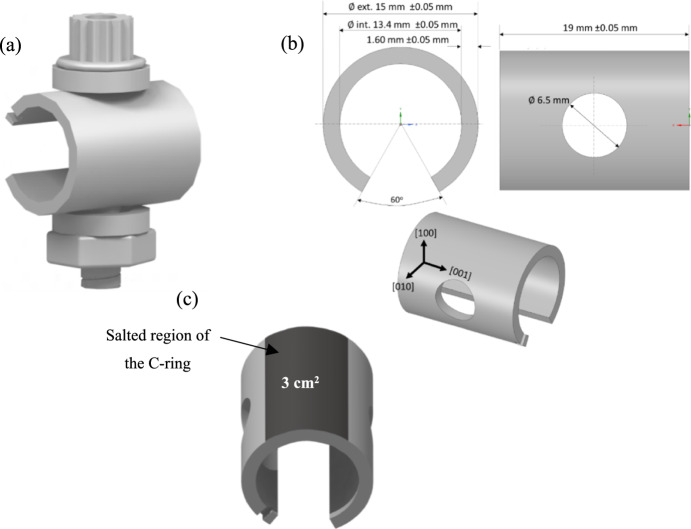
**a** Schematic drawing of C-ring assembly, **b** dimensions of C-ring specimen, **c** region of the C-ring where salt was applied

C-ring test specimens were used throughout this study, produced from CMSX-10 bars in the fully solutioned and aged heat-treated condition as per Rolls Royce Plc. specification with a [001] crystallographic orientation aligned with the cylinder axis, following ISO 7539–5 guidelines. The bolt, washer and nut were made of Waspaloy. The composition of CMSX-10 is listed in Table 1, and the C-ring dimensions are shown in Fig. 1. FEA simulations were undertaken to calculate the required displacement to achieve a target stress level of 450 MPa according to the methodology described in [5]. A jig developed by Rolls Royce Plc. was used as an automated tool to apply the desired displacement on the C-ring specimens as described in [8].

**Table 1:** Nominal chemical composition of CMSX-10 (wt%) [9]

Element	Ni	Al	Cr	Ti	Co	Ta	Re	W	Mo	Hf
Wt%	69.6	5.7	2.0	0.2	3	8.0	6.0	5.0	0.4	0.03

### High-Temperature Exposure

The C-ring specimens were salted with a flux of 0.2 mg/cm^2^ every 50 h on an area of 3 cm^2^ of the C-ring’s apex. This translates to 0.60 mg of salt applied on each specimen every 50 h. The exposure was done in a 50 ppm SO_2_ – air environment at 550 °C and 700 °C. For each salt and at each test temperature, C-rings were exposed for 100 h, 200 h and 500 h as shown in Table 2. Table 3 shows the wt% composition of sea salt used in this study.

**Table 2:** Salts and test conditions used throughout the test

Salt	Exposure time (h)	Temperature (°C)
Sea salt	100, 200 and 500	550 and 700
NaCl	100, 200 and 500	550 and 700
80/20 Na_2_SO_4_/K_2_SO_4_	100, 200 and 500	550 and 700

**Table 3:** Wt % composition of British standard sea salt composition (to DEF 1053/B. S. 3900/B. S. 2011)

Constituent	NaCl	NaHCO_3_	KCl	NaBr	MgCl_2_	MgSO_4_	CaCl_2_
Wt%	76.6	0.58	2.13	0.82	7.01	9.65	3.21

**Fig. 2:**
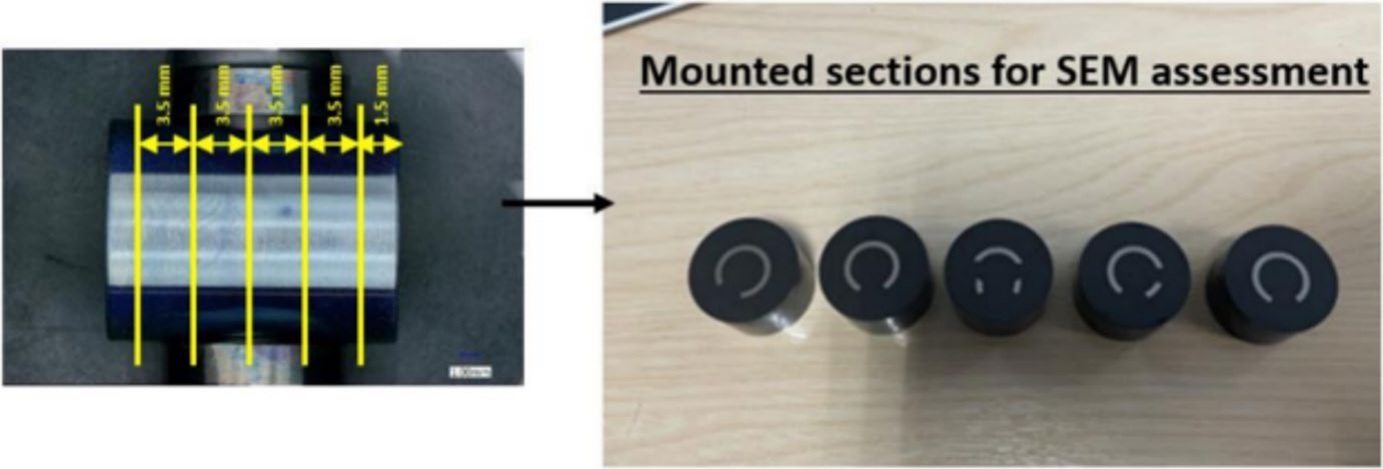
Sectioning locations in C-ring specimen

**Fig. 3:**
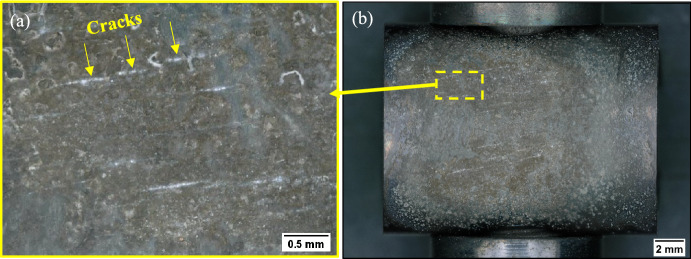
Optical images of the sample salted with NaCl after 3 h of exposure

After each of the exposure times, each C-ring was sectioned in five locations (as shown in Fig. 2), hot mounted, and ground and polished using oil lubricant for crack depth measurements and characterisation of the corrosion products. The crack depth measurements were done on each of the five sections using a Keyence optical microscope, but for specific samples where the cracks were not visible on the optical microscope, backscattered-electron images were used to enable higher magnification images across the circumference of the C-ring. The backscattered-electron images were done using a Tescan s8000 and a beam current of 300 pA (Fig. 3).

## Results

**Fig. 4:**
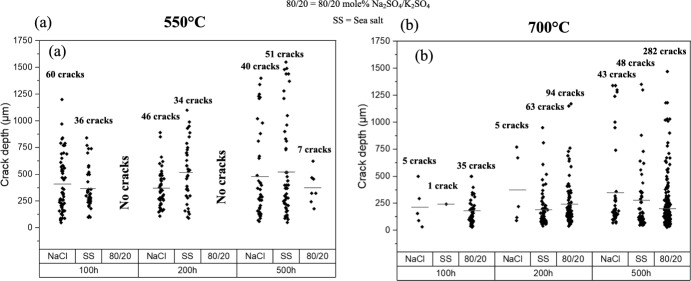
Crack depth measured on C-rings with different salts and exposed at **a** 550 °C and **b** 700 °C

Figure 4 shows the crack depth measured for each of the salted samples at three different exposure times at 550 °C and 700 °C. The results of the 550 °C test show that NaCl and sea salt have crack depths that go almost through the 1.6 mm thickness of the C-ring within the first 100 h and a comparable number of crack initiations at all exposure times. Shorter exposure times were undertaken for the NaCl and sea salted samples and the results showed that stress corrosion cracks initiate in a period between 1 and 3 h of exposure, as shown in Fig. 3 for the NaCl salted sample. For the 80/20 mol% Na_2_SO_4_/K_2_SO_4_, no cracks are observed in the first 200 h of exposure but cracks up to approximately 700 µm are observed after 500 h of exposure. In summary, the test done at 550 °C highlights that chlorine-containing salts lead to cracking in a few hours, whereas 200–500 h is required to crack with the 80/20 mol% Na_2_SO_4_/K_2_SO_4_ salt mix, which represents two orders of magnitude difference in the time to initiate SCC.

The results of the 700 °C test showed a different trend to the 550 °C test. At 700 °C, the 80/20 mol% Na_2_SO_4_/K_2_SO_4_ salted sample showed the highest number of cracks at all exposure times. However, with regard to the crack depth, the 80/20 mol% Na_2_SO_4_/K_2_SO_4_ only shows a slightly higher crack depth after 200 h and 500 h of exposure compared to the NaCl and sea salt salted sample. Also, there is a significantly higher number of cracks observed at 500 h compared to 100 h for the three salted samples.

### Tests Undertaken at 550 °C

**Fig. 5:**
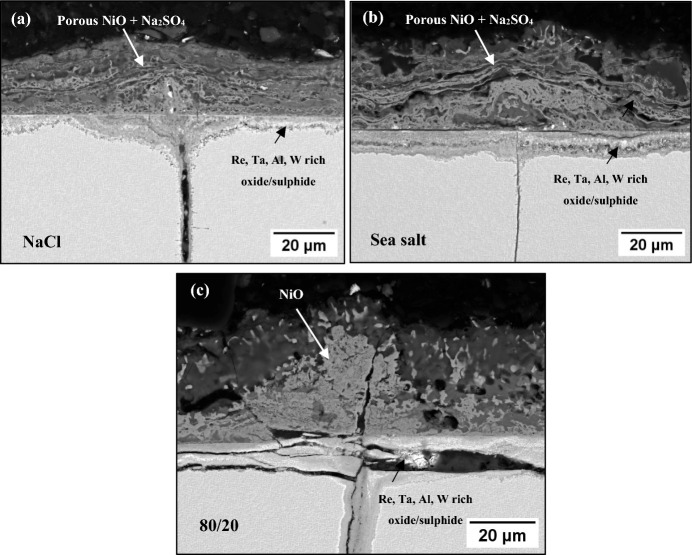
BSE images of NaCl, SS and 80/20 salted samples exposed at 550 °C for 500 h

**Fig. 6:**
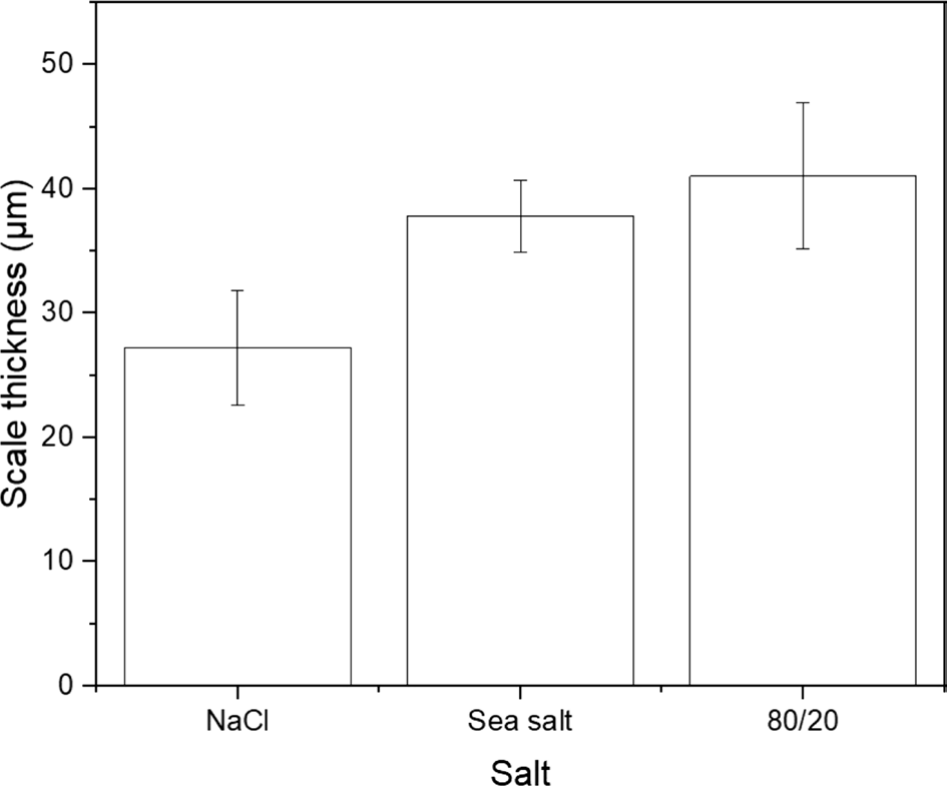
Scale thickness of samples salted with NaCl, sea salt and 80/20 Na_2_SO_4_/K_2_SO_4_ and exposed to 50 ppm SO_2_ at 550 °C for 500 h

Figure 5 shows cross-sectional backscattered-electron images of the salted samples at 550 °C after 500 h. The scale thickness of each sample (shown in Fig. 6) was determined by taking five equidistant measurements of the external and internal scale thickness and averaging the values. (The images used for the measurements are the ones shown in Fig. 5.) The results show that at 550 °C the scale thickness of the 80/20 mol% Na_2_SO_4_/K_2_SO_4_ was slightly higher than the NaCl and sea salt salted samples, which is an interesting observation as that salt showed the lowest susceptibility to SCC.

#### NaCl and Sea Salt at 550 °C

**Fig. 7:**
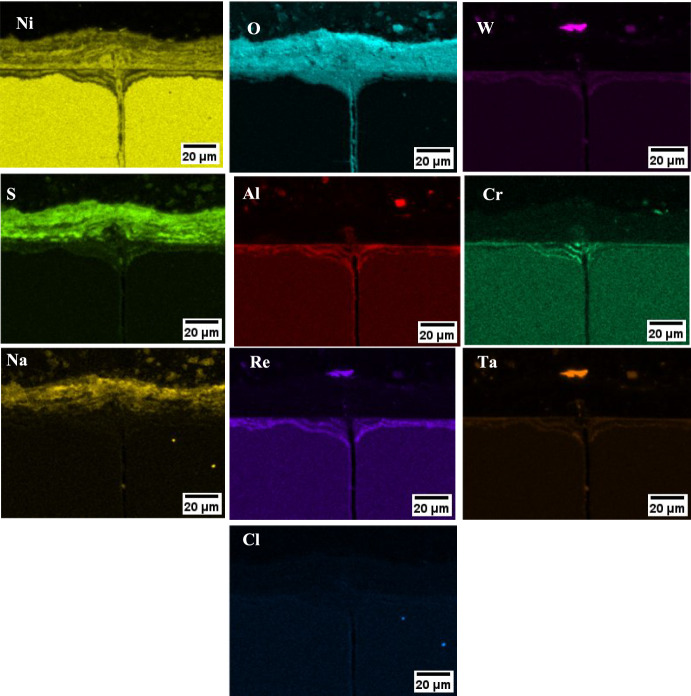
**a** EDS map of NaCl at 550 °C for 500 h, **b** schematic of corrosion products observed

Based on the EDS map of the NaCl salted sample (in Fig. 7), the outer scale on the chlorine-containing salts is composed of a porous nickel oxide, followed by an Al, Cr, Re, Ta and W inner scale rich in O and S. The Na associates strongly with the S map, but does not associate with the Cl map, which suggests that most of the NaCl initially deposited has been converted to Na_2_SO_4_.

#### 80/20 mol% Na_2_SO_4_/K_2_SO_4_

**Fig. 8:**
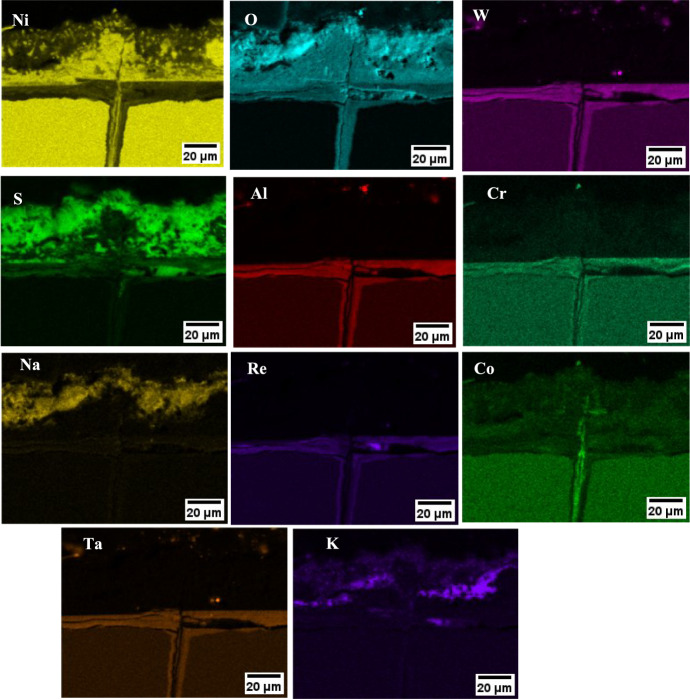
**a** EDS map of 80/20 Na_2_SO_4_/K_2_SO_4_ at 550 °C for 500 h, **b** schematic summary of corrosion products observed

For the case of the 80/20 mol% Na_2_SO_4_/K_2_SO_4_ salted sample, significant concentrations of NiO were observed in the external scale and a mixed oxide/sulphide inner scale rich in Al, Cr, Re, Ta or W was formed as shown in Fig. 8.

The cracks seem to be heavily corroded with sulphate corrodent. This may have occurred due to melt infiltration into the crack, in the case that low melting point eutectics were formed. Also, salt is applied every 50 h of exposure while the samples are still under stress, so it is a possibility that the salt has infiltrated in the crack during the resalting process at room temperature.

### Tests Undertaken at 700 °C

**Fig. 9:**
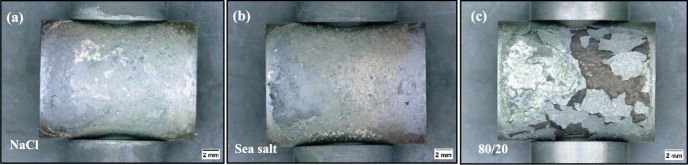
Optical microscope images of the C-ring's top surface for **a** NaCl, **b** sea salt and **c** 80/20 mol % Na_2_SO_4_/K_2_SO_4_ after 100 h of exposure at 700 °C

As mentioned in the results section, the crack depth of the samples tested at 700 °C showed a different trend to those observed at 550 °C in terms of the SCC susceptibility as shown in Fig. 4b. The 80/20 salted sample showed a significantly higher number of cracks at all exposure times compared to the NaCl and sea salt salted samples; however, the maximum crack depth was only slightly higher after 200 and 500 h of exposure. This latter observation is interesting, as it suggests that the different deposit chemistries may affect the number of crack initiation sites, but may not have a significant influence on the crack propagation rate. If corrosion still plays a role in the crack propagation process, this result highlights that the gaseous atmosphere may be the key driver for the crack propagation (Fig. 9).

**Fig. 10:**
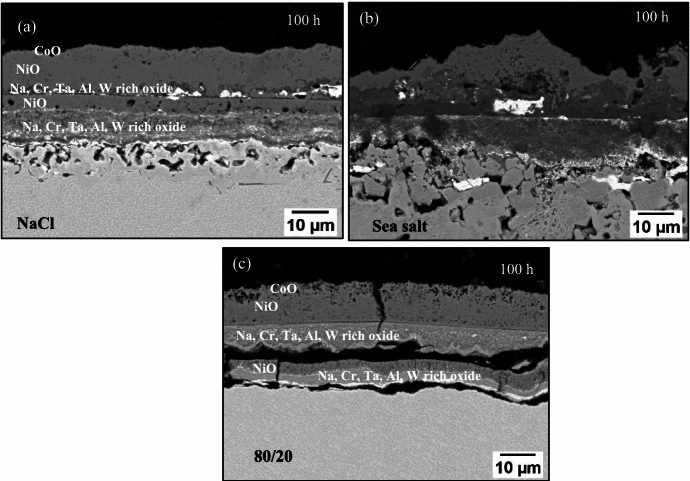
BSE images of samples exposed at 700 °C for 100 h and salted with **a** NaCl, **b** sea salt, **c** 80/20 Na_2_SO_4_/K_2_SO_4_

**Fig. 11:**
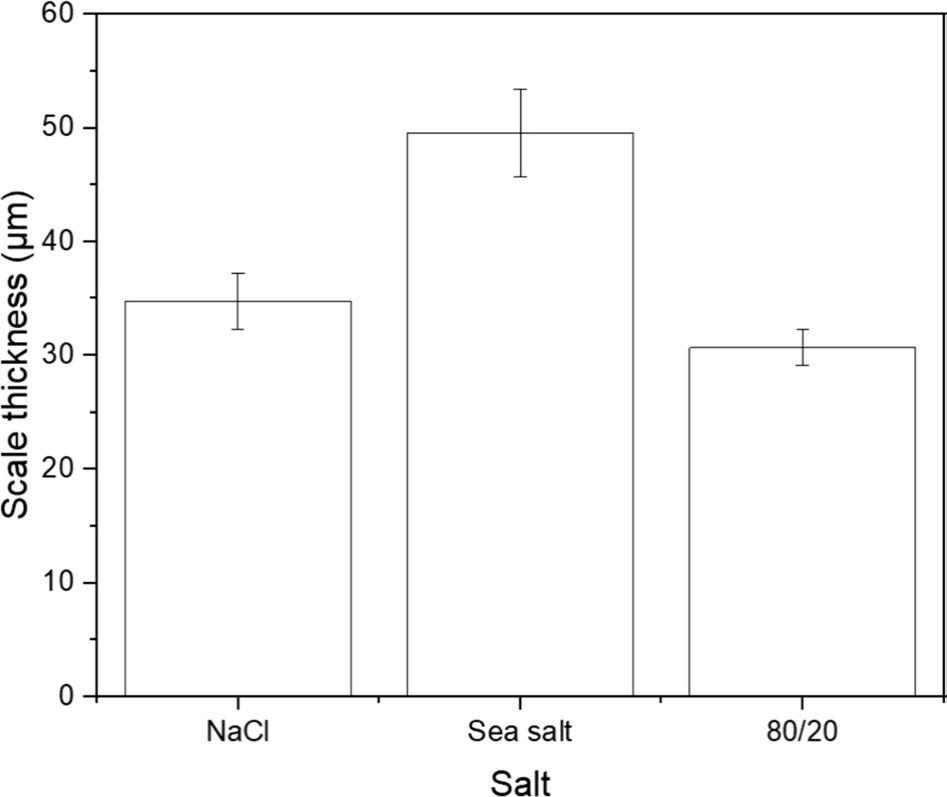
Scale thickness (internal + external) of samples salted with NaCl, sea salt and 80/20 Na_2_SO_4_/K_2_SO_4_ and exposed to 50 ppm SO_2_ at 700 °C for 100 h

According to the BSE images in Fig. 10 and the scale thickness measurements of Fig. 11, the scale thickness was slightly higher for the chlorine-containing salted samples than for the 80/20 salted sample after 100 h of exposure; however, there was significant scale spallation throughout the test, particularly for the 80/20 salt as shown in Fig. 9, which makes the comparison challenging in terms of corrosion damage.

#### NaCl and Sea Salt

**Fig. 12:**
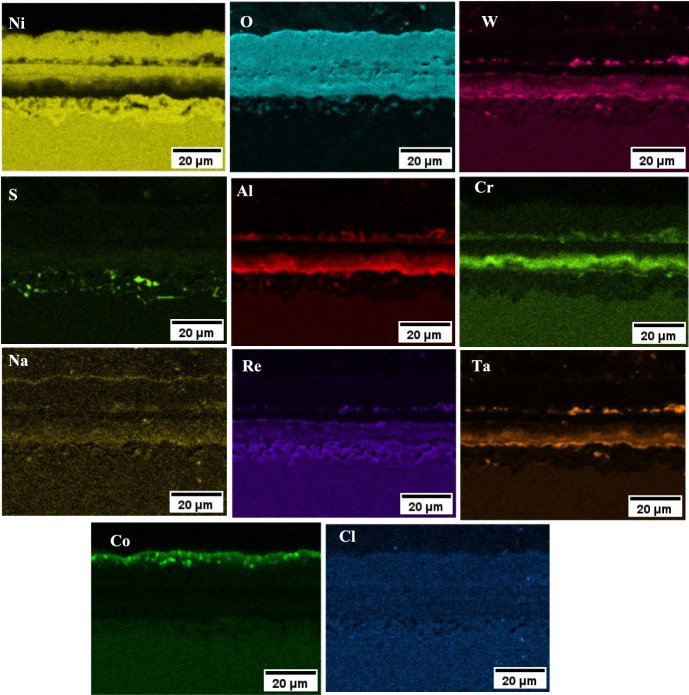
EDS map of NaCl salted sample exposed to 50 ppm SO_2_—air at 700 °C for 100 h

The scale on the NaCl and sea salt salted samples showed a CoO rich layer in the outermost part of the scale, followed by NiO, then an Al, Na, W and Ta rich inner layer, as shown in the EDS map of Fig. 12. The layering repeats itself, possibly due to the lack of scale adhesion at the inner scale-alloy interface, which has led to an increase in the pO_2_ locally. That would explain further formation of NiO, then Al, Cr and Na, W and Ta rich oxide. The EDS map of the NaCl sample also showed S rich particles in the inner scale, which primarily associate with W and Ta. A similar observation was made on the sea salt salted sample, but these S rich particles were absent in the 80/20 sample. Overall, the EDS map of the NaCl salted sample (in Fig. 12) showed a similar scale composition to the sea salt salted sample.

#### 80/20 mol% Na_2_SO_4_/K_2_SO_4_

**Fig. 13:**
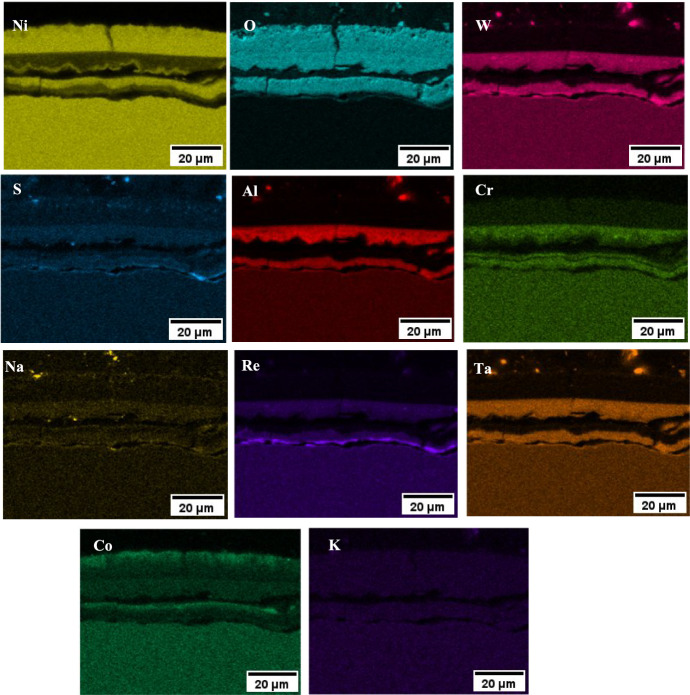
EDS map of the sample salted with 80/20 Na_2_SO_4_/K_2_SO_4_ after 100 h of exposure at 700 °C

The sample salted with 80/20 mol% Na_2_SO_4_/K_2_SO_4_ showed the highest susceptibility in terms of the number of stress corrosion cracks at all exposure times. The EDS map shown in Fig. 13 showed a similar scale composition to the NaCl and sea salt salted samples, where an external layer rich in CoO formed, followed by NiO and ultimately, an Al, Cr, Na, W and Ta rich oxide.

## Discussion

### Test Undertaken at 550 °C

The results of the test undertaken at 550 °C have shown that the time required to initiate stress corrosion cracks in the chlorine-containing salts is approximately 2–3 h, whereas for the 80/20 mol% Na_2_SO_4_/K_2_SO_4_ salt it is between 200 and 500 h according to Figs. 3 and 4b. The reason for this significant difference in behaviour can be attributed to the different corrosion mechanisms for the chlorine-containing salted samples compared to the 80/20 mol % Na_2_SO_4_/ K_2_SO_4_ salted sample. For the chlorine-containing salts, NaCl and other alkali chlorides present in sea salt (MgCl_2_ and KCl) react with SO_x_ to form their corresponding sulphates (Na_2_SO_4_, MgSO_4_ and K_2_SO_4_) releasing HCl as proposed by Duarte et al. [4] (also shown in Eq. 1 calculated using FactSage 8.2), and this conversion occurs above 330 °C [10].

1 $$2{\mathrm{NaCl}}\left( {\mathrm{g}} \right) + {\mathrm{SO}}_{2} \left( {\mathrm{g}} \right) + 0.5{\mathrm{O}}_{2} \left( {\mathrm{g}} \right) + {\mathrm{H}}_{2} {\mathrm{O}} \to {\mathrm{Na}}_{2} {\mathrm{SO}}_{4} + 2{\mathrm{HCl}}\;\;\; \Delta G_{{550^{^\circ } {\mathrm{C}}}} = - 299202.0\;{\mathrm{J}}$$

This is evidenced by the layer of Na_2_SO_4_ that forms in the outermost part of the scale. Based on further results from Duarte et al. [4], the released HCl reacts with Al, Cr and Ti and is transported outward as volatile AlCl_3_, CrCl_3_ and TiCl_4_, which then oxidises at higher P(O_2_), and releasing HCl again. This mechanism is known as active oxidation and typically leads to porous and non-adherent oxides [11]. Duarte et al. also proposed low melting point eutectics to form due the interaction of NaCl with metal chlorides (i.e. NaCl-AlCl_3_ and NaCl-CrCl_2_), which can ultimately accelerate the corrosion process [4]. The poorly protective oxides facilitate the inward diffusion of SO_x_ and O_2_ to form a mixed oxide/sulphide layer.

There is a difference in the morphology of NiO in the external scale between the chlorine-containing salts and the 80/20 mol% Na_2_SO_4_/K_2_SO_4_ salt mix, which highlights that the outward transport mechanism of Ni may be different. The accelerated corrosion for the 80/20 mol% Na_2_SO_4_/K_2_SO_4_ salted sample cannot be explained due to the formation of a low melting point eutectic as the melting point of pure Na_2_SO_4_ is 884 °C and of K_2_SO_4_ it is 1069°. In addition, the lowest melting point for the Na_2_SO_4_-K_2_SO_4_ system is 830 °C, and although reactions with NiO can further reduce the melting point to as low as 596 °C as suggested by Wang et al. [12], these temperatures are still higher than the test temperature and cannot explain the accelerated corrosion observed. An alternative mechanism that may explain the accelerated outward transport of Ni may be the solid-state mechanism (which does not rely on the formation of a melt) suggested by Kistler et al. [13]. The mechanism involves the formation of a Na_2_Ni_2_SO_5_ at the grain boundaries of Na_2_SO_4_ which promotes outward diffusion of Ni through the deposit. This mechanism has been shown using both Na_2_SO_4_ and K_2_SO_4_, so potentially the solid-state mechanism may be responsible for the accelerated outward diffusion of alloy elements and the possible breakdown of the normally protective oxide observed with the 80/20 mol% Na_2_SO_4_/K_2_SO_4_ salt mix.

It is proposed that the breakdown of the oxide with the chloride salts happens more rapidly compared to the sulphate salt and this may explain the early crack formation in the presence chlorine. For the chlorine-containing salts, high concentrations of HCl are released in the early stages of the exposure leading to the active oxidation mechanism and a non-protective scale [11]. The lack of oxide protection then enables the ingress of SO_x_ and internal sulphidation in the very early stages of the exposure [4, 14]. Sulphidation may be a key player in the embrittlement process, as will be described shortly. In contrast to that, the breakdown of the oxide with the 80/20 mol% Na_2_SO_4_/K_2_SO_4_ salt (due to the solid-state mechanism) happens at a much lower rate, which may explain the delay in crack formation compared to chloride salts.

Although the mechanism of crack formation is not well understood, three theories have been postulated. The first theory is the one proposed by Dawson et al. [14] who observed WS_2_ (metal dichalcogenides) forming in the inner scale. The metal dichalcogenides may serve as electro-catalytic sites for H_2_O splitting [15], where residual moisture is present throughout the test. So, a possible theory is that the formation of hydrogen (facilitated by metal dichalcogenides) can lead to hydrogen embrittlement, but further work is required to prove this theory.

The second theory is that the interaction of S with the alloy has a deleterious effect on the mechanical properties of the substrate and may be the primary reason for cracks initiating. This observation is in line with previous work by Woodford et al. [16], who proposed that the interaction of sulphur with polycrystalline Ni alloys during sulphidation can lead to significant losses in ductility, and this embrittlement effect has proved to be critical in the 500–900 °C temperature range [16]. It is further corroborated by Aghion et al. [17], who undertook fatigue tests of coated polycrystalline nickel-based superalloys at 650 °C in an Ar + 0.05% H_2_S environment and showed significant loss in fatigue life compared to both argon and air exposures. The Ar + 0.05% H_2_S atmosphere caused accelerated sulphidation, and they attributed the reduction in fatigue life to the formation of nickel–nickel sulphide eutectic phases (which have a melting point in the range 625–645 °C) as well as embrittlement caused by elemental sulphur diffusion ahead of the crack tip. The low melting point eutectic of the nickel-nickel sulphide phases is above the test temperature of 550 °C so we would not expect these phases to be the dominant mechanism of embrittlement in our study, but the effect of elemental sulphur diffusion ahead of the crack tip may play a role in this mechanism and should be further studied. These studies were done on polycrystalline Ni alloys (where grain boundaries play an important role in the SCC mechanism) and future work should be done to understand the effect of sulphur in single-crystal nickel-based superalloys.

The third theory is proposed by Duarte et al. suggesting that the release of HCl in the chlorine-containing salts may play a crucial role in accelerating the time required to initiate stress corrosion cracks. Historically the effect of hydrogen halides (e.g. such as HCl) has been widely explored in Ti alloys, where the formation of metal chlorides leads to the release of hydrogen at the alloy/scale interface [18]. Hydrogen embrittlement has been the mechanism proposed for stress corrosion cracking in Ti alloys even at temperatures up to 500 °C. Clearly, this study suggests that metal chlorides form in single-crystal nickel-based superalloys in the presence of NaCl and SO_x_; however, the effect of the released hydrogen at the alloy/scale interface is not understood and should be further investigated.

In summary, it is proposed that the breakdown of the protective oxide caused by the 80/20 mol% Na_2_SO_4_/K_2_SO_4_ salt mix at 550 °C is caused by a solid-state mechanism as proposed by Kistler et al. [13]. What makes chlorine so deleterious is that the breakdown of the protective oxide occurs within the first few hours of exposure, whereas for the sulphate-based salts it takes several hundred hours to occur. Ultimately, it seems that the role of the salt is to breakdown the protective oxide, leading to the ingress of SO_x_ and potentially sulphur embrittlement and crack initiation. The embrittlement mechanism may also be caused due to formation of H_2_, as H_2_ may be released when HCl reacts with alloying elements at the scale/alloy interface, but further evidence is required to support this mechanism.

### Test Undertaken at 700 °C

The results of the test undertaken at 700 °C have shown that the crack depth on the 80/20 mol % Na_2_SO_4_/K_2_SO_4_ salted sample is only slightly higher than the chlorine-containing salts only for the 200 h and 500 h exposure, according to Fig. 4b. However, the number of cracks of the 80/20 mol % Na_2_SO_4_/K_2_SO_4_ is higher than the chlorine-containing salts at all exposure times.

Although the reason for this increased number of cracks in the 80/20 salted sample is not entirely understood, a possible explanation is that the 80/20 salt forms eutectics with lower melting points than sea salt and NaCl, which will be further discussed in this section. It is in general agreement that molten deposits have an increased ability in generating severe hot corrosion attack than gaseous or solid salts; so, considering the potential of these three salts of producing a liquid is important. This increased susceptibility to corrosion in the presence of molten salts is because of the larger solubility of the oxides (destructive fluxing action of the normally protective oxide [19]), the faster transport of SO_3_ in the liquid phase and the higher reaction kinetics in the presence of a melt [20, 21].

The lower melting point eutectic increases the fraction of liquid in the deposit [21], leading to a wider coverage area where fluxing of the normally protective oxide occurs, exposing these areas of the alloy to sulphidation. Ultimately, this leads to a higher number of locations where stress corrosion cracks initiate. Also, it may explain the increased scale spallation observed on the 80/20 salted sample after 100 h of exposure, shown in Fig. 8. This result is different to the observations at 550 °C discussed earlier, where the stress corrosion cracking susceptibility is dependent on the formation of halides (HCl).

#### NaCl and Sea Salt

**Fig. 14:**
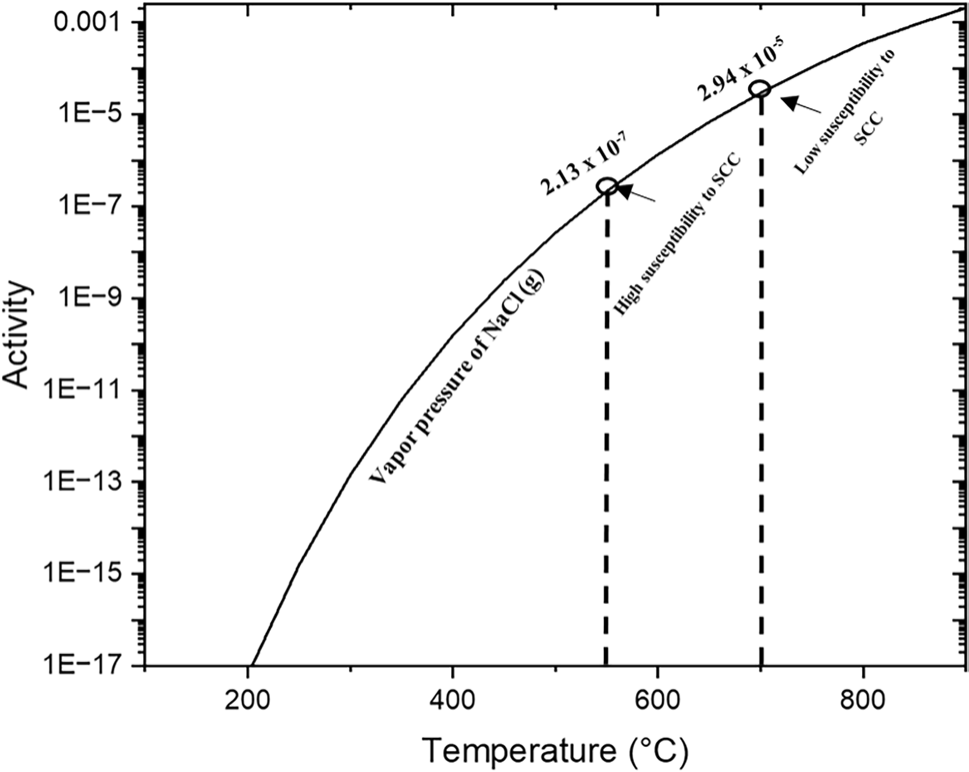
Activity of NaCl (g) in the 200–900 °C temperature range

For the case of the chlorine-containing salts, an important fact to mention is that the vapour pressure of NaCl (g) increases with temperature as shown in Fig. 14; consequently, the increased volatilisation of the salt will have a reduced effect on its susceptibility to SCC compared to its effect at 550 °C.

There are two main interactions that can lead to the formation of low melting point eutectics in the NaCl salted sample. The first one is the possible formation of Na_2_WO_4_, highlighted by the association of Na and W in the inner scale shown in Fig. 12, that has a melting point of 698 °C [22]. This is just below the test temperature and may be involved in accelerating corrosion by an acidic fluxing mechanism. Nevertheless, further work should be done to confirm the formation of Na_2_WO_4_, as other elements such as Al, Cr, Ta and Re are also strongly present in this region.

**Fig. 15:**
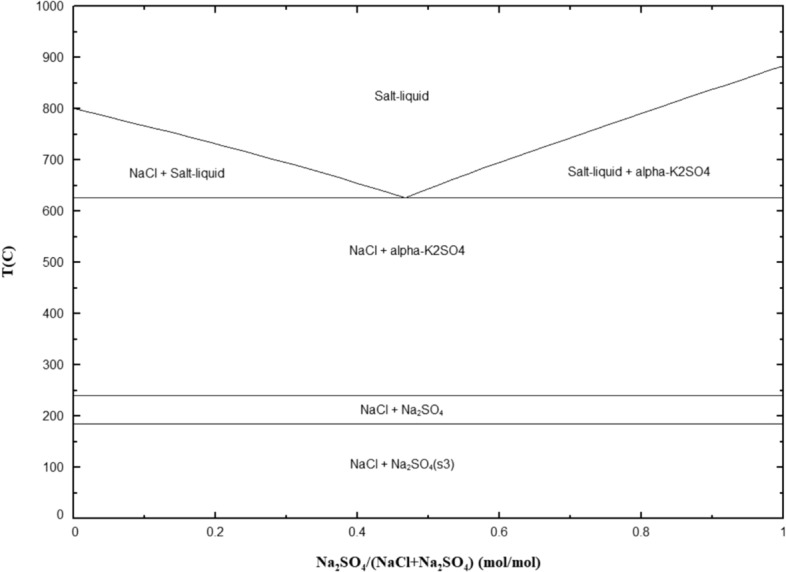
Binary phase diagram of the NaCl–Na_2_SO_4_ system generated with FactSage 8.2 (data from FT salt—Fact salt database)

In addition, the interaction of Na_2_SO_4_ with excess NaCl on the surface can also lead to the formation of a low melting point of 625 °C according to Fig. 15 [23]. However, this possibility only exists in the initial stages of the exposure because after some time most of the NaCl will disappear due to its reaction with SO_x_ mentioned above, possibly leaving a low amount of NaCl available after a few hours of exposure. According to Fig. 15, the interaction of Na_2_SO_4_ with a small amount of NaCl will produce mainly a solid phase with a small proportion of liquid. Similarly, the formation of volatile metal chloride compounds can further lower the melting point of the deposit (only during the initial stages of the test) as well as forming porous oxides and exposing the alloy to the environment [23]. The formation of these liquid phases could explain the fluxing of the protective oxide in the early stages of the exposure that leads to accelerated sulphidation. For comparison purposes, Table 4 summarises the low melting point eutectics that can form with each of the tested salts.

**Table 4:** Low melting point for salt compounds

Salt compound	Systems	Low melting point (°C)
NaCl	NaCl–Na_2_SO_4_	625
	Na_2_WO_4_	698
Sea salt [28]	Na_2_SO_4_–MgSO_4_	666
	NaCl–Na_2_SO_4_	625
	Na_2_WO_4_	698
80/20 Na_2_SO_4_/K_2_SO_4_ [26]	Na_2_SO_4_–K_2_SO_4_	830
	Na_2_WO_4_	698
	Na_2_SO_4_–K_2_SO_4_–NiO–SO_3_	596

Eventually, Na_2_SO_4_ will also be formed during the test, and further work should be done in understanding the sole effect of Na_2_SO_4_ on the corrosion attack (in the absence of NaCl) in the 50 ppm SO_2_ gas environment used throughout this test. It should be highlighted that no liquid phase should be expected with Na_2_SO_4_, as the P(SO)_3_ used in this test (which is approximately 10^–4.5^ atm) is below that required to stabilise the Na_2_SO_4_ – NiSO_4_ liquid phase (with melting point of 671 °C), and the stable phases predicted are solid Na_2_SO_4_ (s)–NiO (s) [13]. Nevertheless, as mentioned earlier Kistler et al. [13] proposed a mechanism of solid-state hot corrosion caused by Na_2_SO_4_ below the eutectic temperature of the Na_2_SO_4_–NiSO_4_ system that accelerates corrosion attack; therefore, it is possible that accelerated corrosion may be partly caused by Na_2_SO_4_ in the solid-state hot corrosion regime throughout this test, but further investigations are required to confirm this.

Sea salt showed a very similar behaviour to NaCl in terms of the number of stress corrosion cracks initiated. Given that 75 wt% of the constituents in sea salt is NaCl, we would expect similar low melting point eutectics to form as the ones mentioned in the NaCl section. In addition, given that sea salt contains a relatively high concentration of MgSO_4_ (approximately 9 wt %), it is important to consider the low melting point eutectic in the Na_2_SO_4_–MgSO_4_ system, which is approximately 666 °C [24, 25], and below the test temperature. However, the formation of the low melting point eutectic in the Na_2_SO_4_–MgSO_4_ system requires approximately 60 wt% MgSO_4_ (higher than what is present in sea salt) so we would mainly expect a solid phase to form in our system, and perhaps liquid phases in local regions of the deposit (where local variations in composition are present). It is acknowledged that other sulphate salts and impurities present in sea salt may contribute to further lowering the melting point as highlighted by Shifler et al. [21], and although it is outside the scope of this study, further work should be undertaken to investigate the effect of these impurities.

#### 80/20 Na_2_SO_4_/K_2_SO_4_

The accelerated stress corrosion cracking attack can be explained by the low melting point eutectics that form below the test temperature. The melting point of pure Na_2_SO_4_ and of K_2_SO_4_ is 884 °C and 1069 °C, respectively, and the lowest melting point of the Na_2_SO_4_-K_2_SO_4_ system is 830 °C [26], which is above the 700 °C test temperature in this study. However, as NiO forms and interacts with the deposit, the system K_2_SO_4_-NiO-SO_3_ may lower the melting point to 642 °C at a P(SO_3_) of 10^–5.3^ atm, and the Na_2_SO_4_-K_2_SO_4_-NiO-SO_3_ system has an even lower melting point of 596 °C at a P(SO_3_) of 10^–5.5^ atm, as shown in Table 4 and as suggested by Wang et al. [12]. The Na_2_SO_4_-K_2_SO_4_-NiO-SO_3_ system gives rise to the lowest melting point identifiable from the three salts studied and may explain its increased susceptibility to SCC compared with NaCl and sea salt. Also, the fact that Ni is involved in the formation of the melt, and the fact that it is the primary constituent in CMSX-10 may explain the increased volume fraction of the liquid phase within the deposit. The increased volume fraction of liquid phase may increase the coverage area where fluxing of the protective oxide occurs, accelerating sulphidation and leading to a higher number of stress corrosion cracks at all exposure times.

These results highlighting the role of K_2_SO_4_ on accelerating corrosion correlate well with studies done by Wang et al. [26] and Shi et al. [27]. Shi et al. [27] observed significant increases in corrosion rates when adding K_2_SO_4_ to Na_2_SO_4_ compared to only Na_2_SO_4_ on Fe–Al alloys. The work showed that adding K_2_SO_4_ to Na_2_SO_4_ reduced the activity of Fe_2_(SO_4_)_3_ required to stabilise the liquid melt, which is consistent with requiring a lower critical P(SO_3_) for the formation of the liquid phase. Eventually, this means that the eutectic liquid melt formed earlier than with Na_2_SO_4_ on its own, reducing the incubation period of hot corrosion. Similarly, Wang et al. [26] also observed increasing corrosion rates when adding K_2_SO_4_ to Na_2_SO_4_ on a β-NiAl coating compared to using Na_2_SO_4_ only. They attributed the increase in corrosion rates to the reduction of the melting temperature of the deposit and a reduction of P(SO_3_) required to stabilise the liquid phase in the presence of K_2_SO_4_. Both studies confirm the deleterious effect of adding K_2_SO_4_ to Na_2_SO_4_ on accelerating corrosion rates and may support the observations in this work.

In general, the results in this study suggest that the increased susceptibility to SCC at 700 °C caused by the 80/20 salt mix is due the formation of a lower eutectic melting point that can be produced compared to NaCl and sea salt. The lower melting point of a compound leads to an increased volume fraction of the liquid phase that will form within the deposit and consequently, increases sulphidation rates [21]. There may be other factors that can affect the SCC susceptibility of the different salts, such as the difference in acidity and basicity of the deposit, and future studies should focus on the effect of these factors on SCC.

As a result of the work carried out, it is evident that a salt mixture (containing both chlorides and sulphates) generates cracking agnostic of the temperature at which the test is undertaken. Conversely, chloride containing salts are most detrimental at 550 °C and predominantly sulphate-based salts are most detrimental at 700 °C. It is therefore imperative to consider this as part of any test methodology, as the salt chemistry can influence the validity of the testing. Salt chemistry varies globally; however, a large proportion of airports are located adjacent to or close to coastal locations which means that BS3900 F4 sea salt represents a suitable engineering approximation to the likely exposure species present in service.

## Conclusions

This study has explored the effect of NaCl, sea salt and 80/20 mol% Na_2_SO_4_/K_2_SO_4_ on the stress corrosion cracking susceptibility of CMSX-10 at 550 °C and 700 °C. At 550 °C, chlorine-containing salts (e.g. NaCl and sea salt) show a higher susceptibility to stress corrosion cracking than the 80/20 mol% Na_2_SO_4_/K_2_SO_4_ salt mix. It is hypothesised that the outward vapour phase transport of alloy elements due to chlorine causes the very early breakdown of the oxide, the ingress of sulphur and ultimately, sulphur embrittlement. At 700 °C, the 80/20 mol% Na_2_SO_4_/K_2_SO_4_ salt mix showed the highest susceptibility to SCC, possibly due to its ability form lower eutectic melting points that can be achieved compared with NaCl and sea salt. The formation of a melt is critical as it leads to the destructive fluxing action of the normally protective oxide, exposing the alloy to accelerated oxidation and sulphidation.

## Data Availability

The data that support the findings of this study are openly available in the following Zenodo link: https://zenodo.org/records/16895629?preview=1&token=eyJhbGciOiJIUzUxMiJ9.eyJpZCI6IjM0ZGNhMjQyLTE3Y2YtNGFiMC1iYWIzLTYzNWRmZmI1ZDdkMyIsImRhdGEiOnt9LCJyYW5kb20iOiIyMTNmMGM3NmEyNWY1YjI2NTYyMGE1NTVhY2EwN2U4OCJ9.PML-FFVfAR-0sHmhylp8PSVbAeo4hZESzvZLTUxsHeya_nFjMaFRjDMLVAS04UEPkrMpa9V-KCZ8UpAaAfuuuA].
